# Genome-wide analysis of the switchgrass YABBY family and functional characterization of *PvYABBY14* in response to ABA and GA stress in *Arabidopsis*

**DOI:** 10.1186/s12870-024-04781-7

**Published:** 2024-02-16

**Authors:** Weiwei Wang, Jiayang Ma, Hanxi Liu, Zhulin Wang, Rui Nan, Tao Zhong, Mengyu Sun, Shaoyu Wang, Yaxin Yao, Fengli Sun, Chao Zhang, Yajun Xi

**Affiliations:** State Key Laboratory of Crop Stress Biology for Arid Areas, College of Agronomy, Northwest A&F University, Yangling, Xianyang, 712100 China

**Keywords:** Switchgrass, YABBY family, Bioinformatic analysis, Functional characterization, Hormone and stress tolerance

## Abstract

**Background:**

The small YABBY plant-specific transcription factor has a prominent role in regulating plant growth progress and responding to abiotic stress.

**Results:**

Here, a total of 16 *PvYABBYs* from switchgrass (*Panicum virgatum* L.) were identified and classified into four distinct subgroups. Proteins within the same subgroup exhibited similar conserved motifs and gene structures. Synteny analyses indicated that segmental duplication contributed to the expansion of the YABBY gene family in switchgrass and that complex duplication events occurred in rice, maize, soybean, and sorghum. Promoter regions of *PvYABBY* genes contained numerous cis-elements related to stress responsiveness and plant hormones. Expression profile analysis indicated higher expression levels of many *PvYABBY* genes during inflorescence development and seed maturation, with lower expression levels during root growth. Real-time quantitative PCR analysis demonstrated the sensitivity of multiple *YABBY* genes to PEG, NaCl, ABA, and GA treatments. The overexpression of *PvYABBY14* in *Arabidopsis* resulted in increased root length after treatment with GA and ABA compared to wild-type plants.

**Conclusions:**

Taken together, our study provides the first genome-wide overview of the YABBY transcription factor family, laying the groundwork for understanding the molecular basis and regulatory mechanisms of *PvYABBY14* in response to ABA and GA responses in switchgrass.

**Supplementary Information:**

The online version contains supplementary material available at 10.1186/s12870-024-04781-7.

## Background

Due to their sessile characteristics, plants are often exposed to various abiotic stresses, such as drought, salt, and cold [1, 2]. Transcription factors play significant roles in the process of plant growth, development, and responses to abiotic stress [3, 4]. The YABBY transcription factor family, a small and specific group within the zinc-finger superfamily, consists of two conserved domains: the N-terminal C2C2 zinc-finger domain and the C-terminal YABBY domain (helix-loop-helix motif) [5, 6]. YABBY transcription factors play important regulatory roles in the establishment of adaxial-adaxial polarity, lamina expansion, and floral organ development [7–9].

YABBY families have been identified and analysed at the genome level in various plant species, including 6 YABBY members in *Arabidopsis thaliana* [10], 8 in rice (*Oryza sativa* L.) [11], 13 in maize (*Zea mays* L.) [12, 13], 21 in wheat (*Triticum aestivum* L.) [12, 14], 17 in soybean (*Glycine max* L.) [15]. In *Arabidopsis*, six YABBY members, including *FILAMENTOUS FLOWER* (*FIL/YAB1*), *YAB2*, *YAB3*, *INNER NO OUTER* (*INO*), *YAB5* and *CRABS CLAW* (*CRC*), were identified and classified into five subfamilies [10]. Generally, *FIL*, *YAB2*, *YAB3* and *YAB5* participate in the formation of vegetative tissues and the development of floral organs. The expression of *INO* and *CRC* was limited to the reproductive organs, ovule, carpel, and nectary development [16–18]. *OsYABBY1*, a gene expressed in stamen, carpel primordium, and meristem, was found to be involved in the feedback regulation of GA biosynthesis, resulting in a semidwarf phenotype in the overexpression lines [11, 19]. *DL*, the homolog of *AtCRC*, plays a role in regulating carpel development and leaf midvein formation [20]. *The OsYABBY4* gene, a member of the FIL/YAB1 group, plays critical roles in meristem and phloem tissue development and regulates plant height through the GA signaling pathway [21, 22]. In maize, the expression of *zyb9* and *zyb14* is limited to the adaxial side, exerting a regulatory effect on blade outgrowth [23]. The *YABBY* gene has been demonstrated to participate in drought, salt, and ABA stress tolerance in soybeans [15]. Additionally, *YABBY* gene plays a negative regulatory role in upland cotton under drought and salt stress [15, 24]. The overexpression of *AcYABBY4* in *Arabidopsis* led to shortened roots under NaCl treatment, indicating a negative regulatory role of *AcYABBY4* in plant resistance to salt stress [25]. While, extensive studies on YABBY transcription factors had primarily focused on vegetative and reproductive processes, their roles in stress and hormone responses in plants are also integral [25, 26]. Switchgrass (*Panicum virgatum* L.), a perennial C4 grass known for its high biomass yield, is considered a model bioenergy crop due to its high yield and low input requirements [27, 28]. Previous research on switchgrass has mainly focused on its physiological and biochemical aspects as a forage or energy crop, with genome-level research still in its infancy. YABBY, as a key transcription factor, has not yet been reported in switchgrass. In this study, we conducted a comprehensive investigation of the YABBY family members in switchgrass at the whole genome level. Subsequently, we analyzed the gene expression profiles of *YABBY* genes in different tissues and under various stresses and hormones. Furthermore, we generated *PvYABBY14*-overexpressing transgenic *Arabidopsis* strains to conduct a detailed functional analysis. This study sheds light on the potential biological functions of the YABBY family members in plant growth and stress tolerance in switchgrass, revealing that *YABBY14* may function as a nuclear transcription factor to regulate plant life processes through ABA and GA signaling pathways.

## Results

### Basic information on switchgrass *YABBY* genes

To identify *YABBY* genes in switchgrass, the hidden Markov model (PF04690) was obtained from the Pfam to search the genome database. A total of 16 YABBY family members were identified, and NCBI CD was used to confirm that these genes had conserved YABBY domains. Each *PvYABBY* gene was assigned a unique name from *PvYABBY1* to *PvYABBY16* based on their genome location in switchgrass (Table 1). The pI and MW of PvYABBY protein sequences are listed in Table 1. The length of the YABBY protein ranges from 162 to 312 amino acid. There were significant variations in MWs; PvYABBY16 had an MW of 17.43 kDa and PvYABBY1 was 32.68 kDa. The isoelectric point ranges from 6.88 to 9.30. The 16 *YABBY* genes were unevenly distributed on 10 chromosomes, including Chr01K, Chr01N, Chr02K, Chr02N, Chr03K, Chr03N, Chr07K, Chr07N, Chr09K, and Chr09N, among which 8 chromosomes had no *YABBY* genes.

**Table 1:** Basic information on PvYABBY members

Serial No.	Gene name	ID	Chromosome	Protein Length(aa)	CDS length(bp)	Isoelectric points (PI)	MW(kDa)	Location
1	PvYABBY1	Pavir.1KG411500.1	Chr01K	264	795	8.13	27.51	43,588,252–43,591,754
2	PvYABBY2	Pavir.1NG370400.1	Chr01N	259	780	8.13	27.29	53,001,536–53,007,132
3	PvYABBY3	Pavir.2KG065516.1	Chr02K	168	507	9.15	18.94	4,837,616–4,843,479
4	PvYABBY4	Pavir.2KG500900.1	Chr02K	162	489	6.88	17.43	61,405,888–61,409,064
5	PvYABBY5	Pavir.2NG043900.3	Chr02N	168	507	9.15	19.01	4,397,909–4,404,910
6	PvYABBY6	Pavir.2NG548700.1	Chr02N	166	501	6.88	17.64	62,876,712–62,879,207
7	PvYABBY7	Pavir.3KG553300.1	Chr03K	198	597	9.3	22.05	62,705,874–62,711,971
8	PvYABBY8	Pavir.3NG273400.4	Chr03N	198	597	8.84	22.24	68,105,549–68,111,954
9	PvYABBY9	Pavir.7KG266200.1	Chr07K	279	840	7.05	29.03	40,872,986–40,875,424
10	PvYABBY10	Pavir.7NG342100.1	Chr07N	277	834	7.58	29.24	39,074,370–39,076,861
11	PvYABBY11	Pavir.9KG067400.3	Chr09K	178	537	9.08	20.14	13,884,482–13,891,948
12	PvYABBY12	Pavir.9KG142600.1	Chr09K	312	939	9.03	32.68	18,984,688–18,989,280
13	PvYABBY13	Pavir.9KG622300.2	Chr09K	195	588	9.01	21.88	63,048,379–63,052,712
14	PvYABBY14	Pavir.9NG181900.1	Chr09N	191	576	9.16	21.42	14,910,092–14,917,705
15	PvYABBY15	Pavir.9NG248800.1	Chr09N	308	927	9.03	32.06	20,691,603–20,696,423
16	PvYABBY16	Pavir.9NG642000.1	Chr09N	197	594	9.01	22.15	74,140,168–74,145,141

### Phylogenetic analysis

**Fig. 1:**
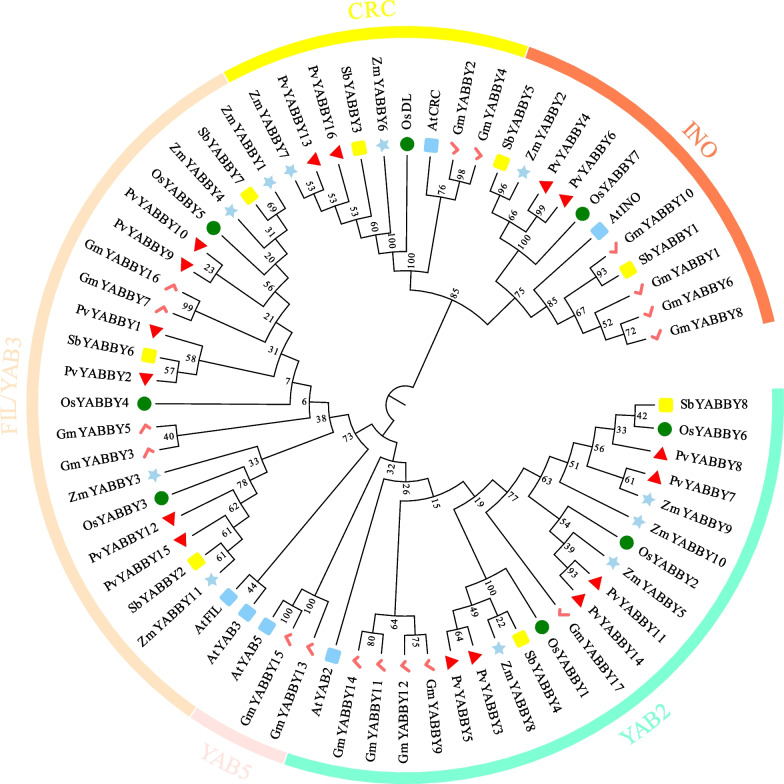
Phylogenetic analysis of the YABBY proteins in *Arabidopsis*, rice, maize, soybean and sorghum. Blue strips represent *Arabidopsis*, green circles represent rice, red checks represent soybean, blue stars represent maize, yellow rectangles represent sorghum, and red triangles represent switchgrass. The outer circle represents five subgroups. Numbers at the nodes indicate bootstrap values

To analyse the phylogenetic relationships of *PvYABBY* genes, an unrooted NJ tree was constructed based on monocotyledonous and dicotyledonous plants of 16 YABBY proteins from switchgrass, 6 from *Arabidopsis*, 8 from rice, 11 from maize, 17 from soybean and 8 from sorghum (Fig. 1, Supplementary Table 1). The results showed that YABBY proteins in the six species were grouped into five subclasses: INO (11 proteins), CRC (9 proteins), FIL/YAB3 (22 proteins), YAB5 (3 proteins), and YAB2 (20 proteins). The YABBY members of switchgrass, maize, rice, and sorghum were distributed among four subclasses: INO, CRC, FIL/YAB3, and YAB2, while only *Arabidopsis* and soybean proteins appeared in the YAB5 subclass, which seems to be a unique subgroup of dicotyledonous plants.

**Fig. 2:**
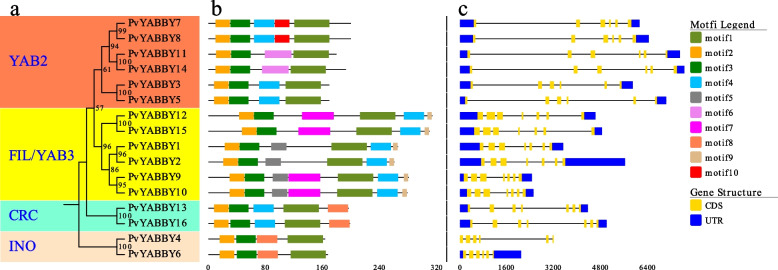
Phylogenetic relationships, conserved motifs, and gene structure analyses of switchgrass YABBY members. **a** The phylogenetic tree was constructed based on the full-length sequences of 16 PvYABBY proteins. The tree shows the 4 phylogenetic subgroups (YAB2, FIL/YAB3, CRC, INO) with 1000 bootstrap values. **b** Nine conserved motifs from YABBY proteins are displayed in different colored boxes. The number below refers to the length of the protein. The sequence logos and E-values for each motif are shown in Supplementary Fig. 1. **c** Exon–intron structure of *PvYABBY* genes. Exons and introns are indicated by yellow boxes and single lines, respectively. Blue boxes represent upstream or downstream UTR regions. The number below refers to the length of the genes

Subsequently, a phylogenetic tree of switchgrass was constructed to determine the phylogenetic relationships of PvYABBY proteins (Fig. 2a). The results showed that the 16 YABBY members could be divided into four subgroups: INO, CRC, FIL/YAB3 and YAB2. The FIL/YAB3 and YAB2 subgroups had six YABBY members each, and the INO and CRC subgroups had two YABBY members each.

### Conserved motifs and gene structure analysis

Protein function and evolutionary relationships are commonly determined by the type and composition of conserved motifs. To identify the conserved motifs of PvYABBY proteins, we analysed 10 motifs (motif 1-motif 10) in the 16 YABBY members of switchgrass using the MEME online program (Fig. 2b). The results showed that the PvYABBY protein typically contains 4–6 motifs, with motif1, motif2, and motif3 being as highly conserved domains present in all YABBY protein sequences. These motifs are considered the YABBY domain, as indicated by the Pfam domain search. Moreover, specific motifs unique to certain subgroups were identified, such as motif4 appears in the CRC, FIL/YAB3 and YAB2 group, motif5, motif7, motif9 are found in the FIL/YAB3 group, motif6 and motif10 are present in the YAB2 group, motif8 is specific to the INO and CRC groups. Overall, YABBY proteins belonging to the same subfamily exhibit similar conserved motif patterns, each with characteristic conserved motifs in different subfamilies.

The divergence in exon–intron structure typically reflects evolutionary information within gene families. The gene structure information of the YABBY members was obtained from the switchgrass genome and visualized using Evolview. As shown in Fig. 2c, the *PvYABBY* genes display varying numbers of introns and exons, indicating that members within the same subgroup share conserved gene structures, while *PvYABBYs* in different subgroups exhibit some differences. For instance, the FIL/YAB3 and CRC subfamilies contain 6 introns, whereas INO and YAB2 contain 5 introns. However, it is noteworthy that the number of introns among members of the YABBY family does not significantly differ and remains relatively conserved during evolution. The slight variation in gene structure among YABBY members suggests their relative conservation during evolution.

### Synteny analysis of *YABBY* genes in switchgrass

**Fig. 3:**
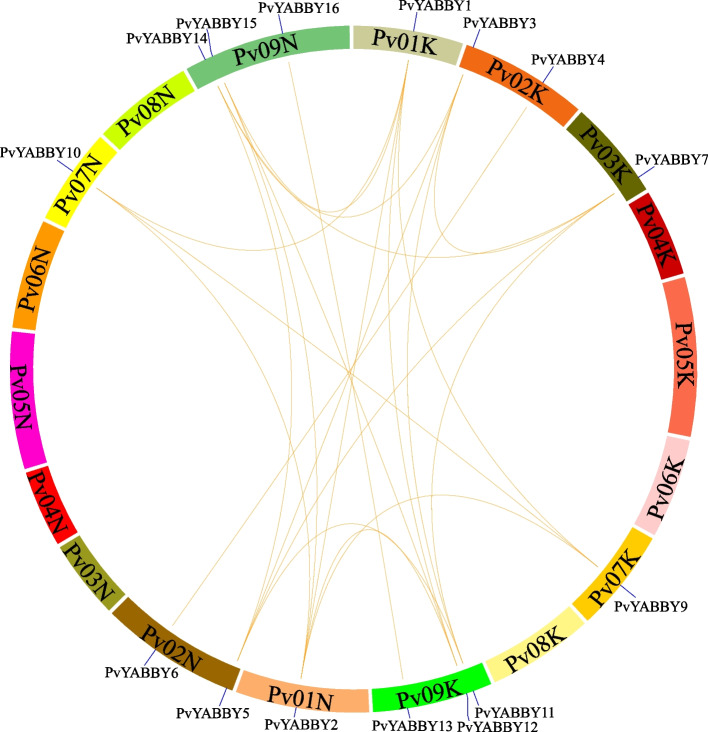
Genomic localization and gene duplication of *PvYABBY* genes. The duplicated gene pairs of *PvYABBY* are connected with orange lines. Eighteen chromosomes were arranged in circles with different colors on the periphery

Synteny analysis was conducted to examine the *YABBY* homologous gene relationships and putative gene duplication events. The results revealed 27 pairs of synteny events were identified in the switchgrass PvYABBY family (Fig. 3, Supplementary Table 2). Most *YABBY* genes were involved in multiple synteny relationships. For instance, *PvYABBY1* showed homology to *PvYABBY2*, *PvYABBY9*, *PvYABBY10*, *PvYABBY12*, and *PvYABBY15*, and *PvYABBY3* was homologous to *PvYABBY7*, *PvYABBY8*, *PvYABBY11*, and *PvYABBY14*. Notably, two homologous gene pairs exhibited one-to-one syntenic relationships: *PvYABBY4* -*PvYABBY6* and *PvYABBY13* - *PvYABBY16*. The duplication patterns of all *PvYABBY* genes arose from segmental duplications without tandem duplications, indicating that segmental duplications likely contributed significantly to the expansion of the switchgrass YABBY family.

### Synteny analysis of *PvYABBY* genes in six species

**Fig. 4:**
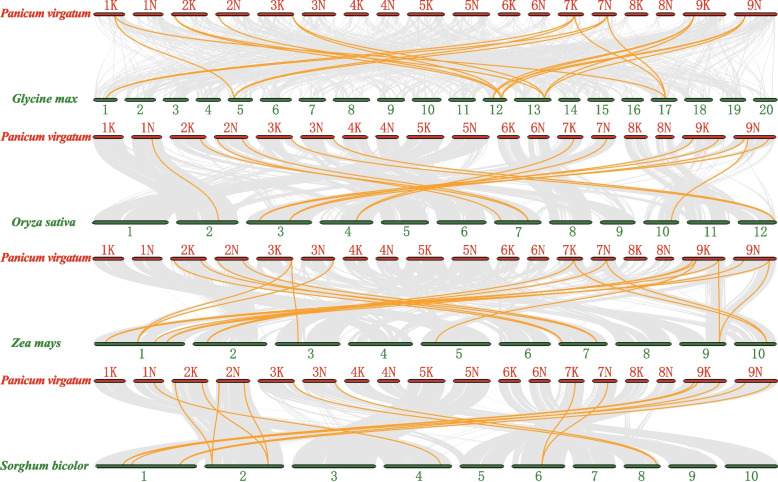
Gene duplication and synteny analysis of *YABBY* genes among switchgrass with *Arabidopsis thaliana*, soybean, rice, maize, and sorghum. No synteny pair was found between *Panicum virgatum* and *Arabidopsis thaliana*. Red and green oval boxes represent the number and length of chromosomes. The duplicated gene pairs of YABBY are connected with orange lines

To further explore the evolutionary relationship of the *PvYABBY* gene family in switchgrass, five comparative syntenic maps were constructed consisting of two dicotyledonous plants (*Arabidopsis* and soybean) and three monocotyledonous plants (maize, sorghum, rice). The numbers of homologous pairs among switchgrass and each of the comparative species were 14, 19, 19, 13, and 0 for soybean, maize, sorghum, rice, and *Arabidopsis*, respectively (Fig. 4, Supplementary Table 3). These syntenic gene pairs showed various corresponding relationships, including one switchgrass gene corresponding to several gene in the comparative species, such as *PvYABBY7*-*ZmYABBY10/ZmYABBY9 and PvYABBY9-GmYABBY3/GmYABBY7/GmYABBY16*, others had multiple switchgrass genes corresponding to one gene in the comparative species, such as *SbYABBY8*-*PvYABBY7*/*PvYABBY8* and *OsYABBY6*-*PvYABBY7*/*PvYABBY8*. Additionally, some relationships showed one gene in switchgrass corresponding to one gene in the comparison species, such as *PvYABBY2*-*SbYABBY6*, *PvYABBY2*-*OsYABBY4*, *PvYABBY5*-*OsYABBY1*, and *PvYABBY15*-*OsYABBY3*. These findings from comparative genomics analysis suggest that these orthologous genes were already present before ancestral divergence.

### Predicted cis-elements in the promoters of *PvYABBYs*

**Fig. 5:**
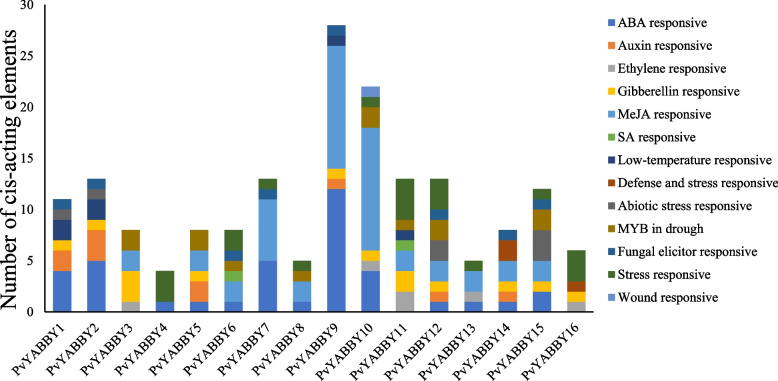
The identification and distribution of the cis-elements related to stress and hormone tolerance derived from *YABBY* promoters

The 1500 bp upstream CDS sequences were extracted from the promoter regions of 16 *PvYABBY* genes. *Cis*-acting elements in the promoter region of *PvYABBY* predicted by the PlantCARE database. A total of 18 cis-elements with clear functions were predicted and revealed in the study (Fig. 5, Supplementary Table 4). These cis-elements were associated to various hormones, including ABA responsiveness (13 *PvYABBYs*), auxin responsiveness (6 *PvYABBYs*), ethylene responsiveness (5 *PvYABBYs*), gibberellin responsiveness (11 *PvYABBYs*), MeJA responsiveness (12 *PvYABBYs*), and SA responsiveness (2 *PvYABBYs*). Additionally, several cis-elements were related to stress responses including low-temperature responsiveness (4 *PvYABBYs*), defense and stress responsiveness (2 *PvYABBYs*), abiotic stress responsiveness (4 *PvYABBYs*), MYB in drought (8 *PvYABBYs*), fungal elicitor responsiveness (8 *PvYABBYs*), stress responsiveness (10 *PvYABBYs*), and wound responsiveness (1 *PvYABBY*). Overall, the results suggest that *PvYABBYs* play crucial roles in responding to hormone treatments, low temperature, drought stress, and other stress-related processes.

### Expression analysis of *PvYABBYs* in different tissues and growth stages

**Fig. 6:**
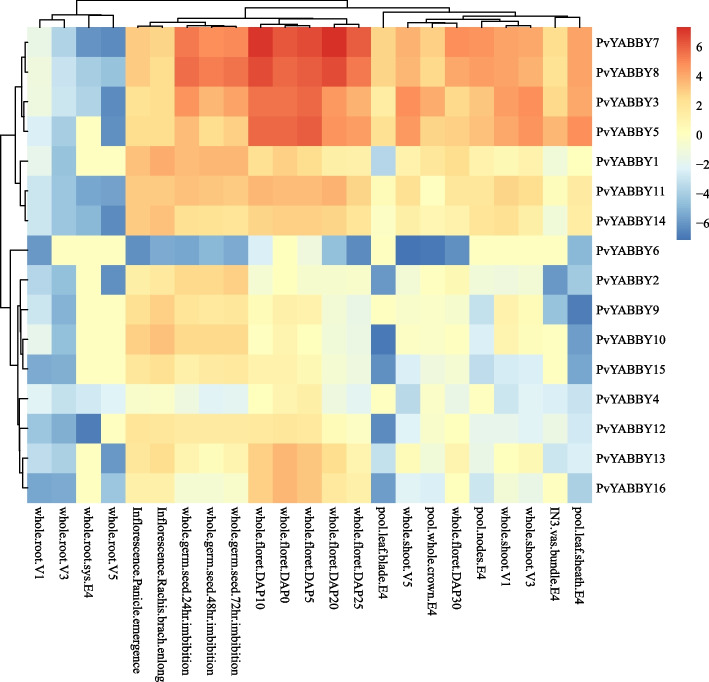
Expression profiles of *PvYABBYs* in different tissues and growth stages. Note: IN3 vas bundle. E4 represents the vascular bundle isolated from the 1/5 fragment of internode 3. These tissues of pool leaf blade. E4, pool leaf sheath. E4, pool nodes. E4, pool whole crown. E4 and whole root sys were collected from the leaf blade, leaf sheath, nodes, and root system in elongation growth stage 4 (4 nodes). DAP0, DAP5, DAP10, DAP20, DAP25 and DAP30 represent the whole floret at 0, 5, 10, 20, 25, and 30 days post fertilization, and V1, V3, and V5 represent vegetative growth stage 1 (1 leaf), vegetative growth stage 3 (3 leaves), and vegetative growth stage 5 (5 leaves)

To explore the potential roles of *PvYABBY* genes in plant growth and development, the expression patterns of *YABBY* genes in 23 tissues were derived from the JGI database and visualized with R (Fig. 6, Supplementary Table 5). The results indicated that most of the *YABBY* genes in switchgrass exhibited lower expression levels in the roots but higher expression levels in the seeds and inflorescence tissues. Further analysis revealed that several *PvYABBYs*, such as *PvYABBY7*, *PvYABBY8*, *PvYABBY3*, and *PvYABBY5,* showed strong induction in inflorescence tissues but lower expression in the roots. Moreover, many *PvYABBYs*, including *PvYABBY1*, *PvYABBY2*, *PvYABBY4*, *PvYABBY9*, *PvYABBY10*, *PvYABBY11*, *PvYABBY12*, *PvYABBY13*, *PvYABBY14*, *PvYABBY15*, and *PvYABBY16*, displayed relatively high expression in both seed and inflorescence tissues but relatively low expression during growth stages. However, *PvYABBY6* exhibited relatively low expression levels across all stages. These tissue-specific expression profiles suggest that *PvYABBYs* likely have distinct functions at different stages of plant growth and development.

### Expression analysis of *YABBY* genes by qRT–PCR under different hormone and stress treatments

**Fig. 7:**
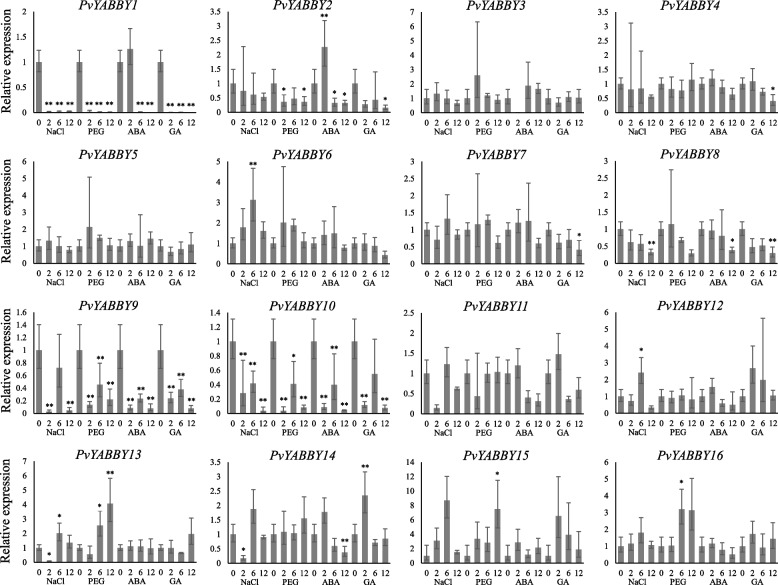
qRT–PCR analysis of *YABBY* genes in switchgrass under hormone and stress treatments. The X-axis represents the hormone and stress processing duration (0, 2, 6, 12 h), and the Y-axis represents the relative expression level. Error bars refer to positive and negative deviations. * refers to *P* value< 0.05, ** refers to *P* value< 0.01

To further investigate the potential roles of *YABBY* genes in switchgrass under hormone and stress treatments, we subjected hydroponic seedlings to drought (PEG), salt (NaCl), ABA and GA treatments. The expression patterns of many *PvYABBY* genes were found to differ under stress or hormone treatments (Fig. 7, Supplementary Table 6). For instance, the expression of *PvYABBY1*, *PvYABBY8*, *PvYABBY9*, *PvYABBY10*, and *PvYABBY14* was significantly repressed after NaCl treatment, whereas the expression of *PvYABBY6* and *PvYABBY12* increased at 24 h, and the expression level of *YABBY12* showed a decrease followed by an increase. Simulating drought treatment revealed reduced expression levels for several *PvYABBY* genes, such as *PvYABBY1*, *PvYABBY2*, *PvYABBY9*, and *PvYABBY10*. Conversely, *PvYABBY13*, *PvYABBY15*, and *PvYABBY16* exhibited decreased expression levels at 6 h or 12 h after PEG treatment. *PvYABBY9* and *PvYABBY10* displayed similar expression patterns after ABA treatment, being significantly repressed at all processing times. *PvYABBY1*, *PvYABBY8* and *PvYABBY14* also showed decreased expression levels at 12 h after ABA treatment, with fold changes of 500.0, 2.5, and 2.6, respectively. *PvYABBY2* increased by 2.3-fold at 2 h of treatment but decreased by 2.9-fold at 6 h and 12 h. In response to GA treatment, seven *PvYABBY* genes were downregulated in response to GA treatment, including *PvYABBY1*, *PvYABBY2*, *PvYABBY4*, *PvYABBY7*, *PvYABBY8*, *PvYABBY9*, and *PvYABBY10*, while only *PvYABBY14* was upregulated at 2 h.

### Subcellular localization analysis

**Fig. 8:**
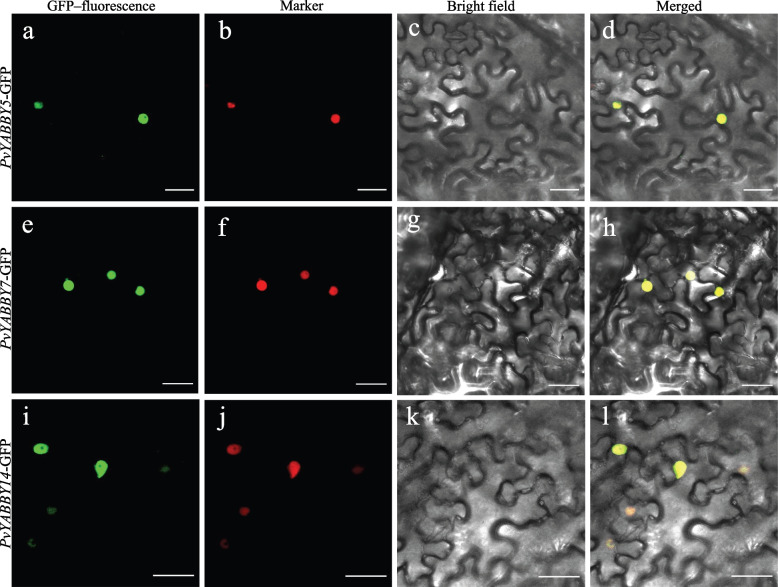
Subcellular localization of *PvYYABYs. PvYABBY5*-GFP, *PvYABBY7*-GFP, *PvYABBY14*-GFP and *nucleus-*mCherry marker were transiently expressed in tobacco epidermal cells, scale bar = 35 μm

To explore the subcellular localization of *PvYABBYs*, we selected *PvYABBY5*, *PvYABBY7*, and *PvYABBY14*, which exhibited distinct expression patterns under hormone and stress treatments, to generate PvYABBYs–GFP fusion proteins. These fusion proteins, along with *nucleus*-mCherry as a positive control, were coinfiltrated into the leaves of *Nicotiana benthamiana* plants. Confocal microscopy was used to visualize the fluorescent signals after two days, revealing that *PvYABBY5*-GFP, *PvYABBY7*-GFP and *PvYABBY14*-GFP were all localized to the nucleus. This localization suggests their potential roles as transcription factors (Fig. 8).

### Phenotypic identification of *PvYABBY14*

**Fig. 9:**
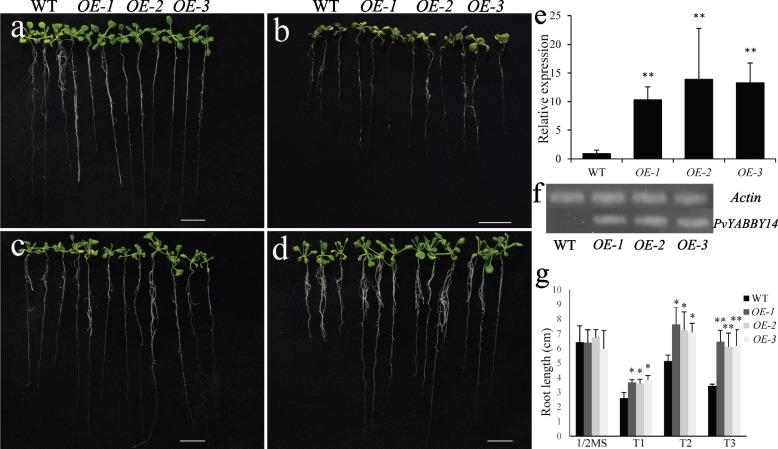
Phenotypic comparison of wild-type (WT) and *PvYABBY14* transgenic *Arabidopsis* under ABA and GA treatments. **a** Seedlings grown on 1/2 MS medium. **b** Seedlings grown on 1/2 MS medium supplemented with 5 μmol·L^−1^ ABA. **c** Seedlings grown on 1/2 MS medium supplemented with 50 μmol·L^−1^ GA. **d** Seedlings grown on 1/2 MS medium supplemented with 100 μmol·L^−1^ GA. **e** Transcript levels of *PvYABBY14* were revealed by qRT–PCR. The bars are the means and standard deviation for each line. **f** Transcript levels of *PvYABBY14* were revealed by seq RT–PCR for three transgenic lines. **g** Root length of wild-type and three transgenic lines under ABA and GA treatments. T1, T2, and T3 represent 5 μmol·L^−1^ ABA, 50 μmol·L^−1^ GA and 100 μmol·L^−1^ GA, respectively. Scale bar = 1 cm. The bars are the means and standard deviation for each line

Numerous *PvYABBYs* harbor multiple cis-elements associated with hormones or stress, a finding corroborated through qRT–PCR analysis. To substantiate the roles of *PvYABBYs* in different plant growth stages and conduct comprehensive functional investigations, *YABBY14* was integrated into an overexpression vector driven by the 35S promoter and subsequently transformed into *Arabidopsis* mediated by *Agrobacterium*. Semiquantitative PCR and qRT–PCR analyses demonstrated elevated transcription levels in three distinct transgenic lines compared to wild-type plants (Fig. 9e, f, Supplementary Fig. 2). No disparities in root length were observed between wild-type and *PvYABBY14* transgenic *Arabidopsis* on 1/2-strength MS media (Fig. 9a, g). However, the root length of transgenic plants was significantly higher than that of wild-type plants when grown on 1/2 MS medium containing 50 μmol·L^−1^ or 100 μmol·L^−1^ GA (Fig. 9c, d, g). Under 5 μmol·L^−1^ ABA treatment (Fig. 9b, g), the root length of transgenic plants surpassed that of wild-type plants by 30%, and there was no difference at a concentration of 20 μmol·L^−1^ (Supplementary Fig. 3a). No noteworthy distinctions were noted between transgenic and wild-type lines treated with 100 mmol·L − 1 and 200 mmol·L − 1 mannitol (Supplementary Fig. 3b, 3c). These findings suggest that *YABBY14* probably modulates plant growth processes through the GA and ABA signaling pathways.

## Discussion

The YABBY family, characterized by the presence of the zinc-finger and YABBY conserved domain, represents a plant-specific transcription factor. These genes exert significant regulatory influence over lateral organ development and the establishment of abaxial-adaxial polarity [8, 10]. Notably, *YABBY* genes are recognized as pivotal contributors to the plant response to abiotic stress [29–31]. Many *YABBY* genes from different species have been identified, including 6 in *Arabidopsis*, 8 in rice, 13 in maize, 21 in wheat, and 17 in soybean. In this investigation, 16 PvYABBY members were identified from switchgrass. Comparatively, the expansion of the switchgrass YABBY family, as observed in this investigation, aligns with an intermediate evolutionary trajectory when contrasted with previous findings.

To elucidate the evolutionary relationships among YABBY members in switchgrass, we constructed a phylogenetic tree incorporating YABBY proteins from four monocotyledons and two dicotyledons. All the YABBY proteins were classified into five clades, with members from *Arabidopsis* and soybean distributed across these subfamilies. Intriguingly, the YABBY members of switchgrass, rice, sorghum, and maize were notably absent in the YAB5 subfamily. This observation suggests a potential reduction in diversity within the YAB5 subfamily during the evolution of monocots, whereas their homologous YAB2 genes are retained in both monocots and dicots. Previous research has proposed that the separation of YAB2 and YAB5 occurred subsequent to the divergence of monocots and dicots, leading to the loss of YAB5 in monocots [32, 33]. The evolutionary patterns observed in switchgrass YABBYs further support this hypothesis.

The results of the phylogenetic analysis indicated that switchgrass YABBY family members could be divided into four subfamilies: INO, CRC, YAB2, and YAB3. Notably, members within the same subgroup, such as PvYABBY4 and PvYABBY6, PvYABBY12 and PvYABBY15, exhibited highly conserved motifs and exon-intron structures. This observation suggests that genes placed within the same subgroup likely share similar functionalities. Conversely, the evolutionary diversity in gene structure implies functional distinctions [34]. For instance, while CRC is implicated in the formation of nectaries and carpels, INO is associated with the development of the abaxial region of the outer integument [10].

The degree of variation in gene structure serves as an indicator of evolutionary conservation within the family. GSDS analysis revealed that the PvYABBY proteins typically harbor five or six introns, a pattern consistent with observations in rice [11], wheat [14], soybeans [15], grapes [35], etc. The conservation of YABBY members during expansion suggests functional similarities. This conservation further implies that the primary function of switchgrass YABBY proteins in growth and development may manifest in the development of floral meristems and lateral organs.

The evolution of plant genomes often involves segmental duplications and tandem duplications, contributing to the expansion of gene families [36, 37]. Recent research has emphasized the significance of segmental duplications in shaping the evolution of the *YABBY* gene family over tandem duplications [38, 39]. In the synteny analysis of switchgrass, 27 segmental repetitive events involving 16 *PvYABBY* genes were identified. To gain further insights into the evolution of YABBY members, we conducted synteny analysis with two dicotyledons and four monocotyledons. All synteny events between switchgrass and rice or sorghum indicated relationships where one switchgrass gene exhibited synteny with one or more rice or sorghum genes. This suggests that these orthologous *PvYABBY* genes are conserved and may have existed before the divergence of their common ancestor. In contrast, 19 synteny events were observed between eight *PvYABBY* genes in switchgrass and six *GmYABBY* genes in soybean, indicating multiple gene expansion events occurred after the divergence of switchgrass and soybean. These duplication events have contributed to an increase in the number of gene family members, preventing the loss of gene function caused by heredity or chromosome mutation [40].

Some homologous genes originating from duplication events have similar expression patterns [41]. A comparison of 27 pairs of synteny events with tissue expression profiles revealed predominant similarities in expression patterns among syntenic gene pairs. Notably, *PvYABBY3*, *PvYABBY5*, *PvYABBY7*, *PvYABBY8*, *PvYABBY11*, and *PvYABBY14* exhibited elevated expression levels during early reproductive stages, while *PvYABBY9* and *PvYABBY10* displayed heightened expression in late reproductive stages and early seed germination. Consistency in expression patterns extended to stress and hormone treatments, as observed through qRT-PCR analysis. Both *PvYABBY9* and *PvYABBY10* demonstrated downregulation under NaCl, PEG, ABA, and GA treatments, whereas *PvYABBY13* and *PvYABBY16* exhibited upregulation under PEG treatment. These consistent expression profiles suggest the retention of similar functions in these gene pairs, indicative of shared ancestry [42]. However, notable differences were identified in some synteny pairs, reflecting potential functional divergence post-duplication. For instance, *PvYABBY4* and *PvYABBY6* displayed distinct expression peaks in early flowering and vegetative stages, respectively. Additionally, *PvYABBY4* was downregulated under GA treatment, while *PvYABBY6* exhibited upregulation under NaCl treatment. These expression disparities underscore the evolutionary process leading to functional diversity among homologous genes [43–45].

Investigating the expression patterns of *YABBY* genes throughout plant growth, development, and stress response holds promise for enhancing switchgrass stress resistance, ultimately aiming to boost biomass production. The cis-elements present in the promoter region play significant roles in regulating gene expression, and their presence can influence the level of gene expression [12]. Searching for conserved cis-acting motifs can be used to predict gene function and potential interactions [46]. To better understand the regulatory relationship of switchgrass *PvYABBYs*, we isolated the 1.5 kb promoter regions of the *PvYABBY* genes and discovered many stress-related and hormone-related plant regulatory elements. Notably, elements responsive to ABA and GA, such as ABRE, GARE-motif, TATC-box, and P-box, were prevalent in the switchgrass YABBY family. Abscisic acid is involved in regulating plant growth and development, osmotic challenges, and defense against adverse environmental factors [38, 47]. Gibberellins (GAs) are known to play an important role in various aspects of plant growth and development, including stem growth and flowering [48]. The abundance of ABA- and GA-responsive elements suggests that *YABBY* genes may intricately regulate switchgrass growth and development through ABA and GA signaling pathways. The expression of switchgrass *YABBY* genes in diverse tissues and growth stages further supports the hypothesis that these genes play pivotal roles in growth regulation. Moreover, the identification of stress-related cis-elements (DRE, MBS, ARE, and TC rich repeats) within *PvYABBY* genes implies potential involvement in responding to abiotic stresses [49–52]. This dual functionality suggests that switchgrass *YABBY* genes may act as central regulators, not only in developmental processes but also in orchestrating responses to environmental challenges.

Afterwards, qRT–PCR was performed to scrutinize the responses of YABBY family members under various hormone or stress conditions, encompassing ABA, GA, NaCl, and PEG. The findings revealed distinctive expression patterns for most *YABBY* genes in response to these treatments. Notably, *PvYABBY1*, *PvYABBY9* and *PvYABBY10* exhibited significant induction under ABA, GA, NaCl, and PEG treatments. These findings strongly indicate the pivotal roles of *PvYABBYs* in hormone and abiotic stress responses. The response of plants to hormone abiotic stress is a complex process that is regulated by different molecular and cellular pathways. The observed responses of *YABBY* genes to different stresses lay a robust foundation for in-depth functional investigations of switchgrass *YABBY* genes. To delve deeper into their potential functions, the *YABBY14* gene was overexpressed in *Arabidopsis* for functional exploration. Notably, *YABBY14* demonstrated reduced sensitivity to ABA stress, suggesting its involvement in inhibiting plant growth through intricate regulatory mechanisms. Consequently, *YABBY14* emerges as a positive regulatory factor influencing plant responses to ABA stress. The observed root elongation in *YABBY14* transgenic *Arabidopsis* plants under GA treatment further implies that the *YABBY14* gene serves as a positive regulator, promoting overall plant growth and development. This holds promise for achieving enhanced biomass production and, consequently, increased economic benefits. Furthermore, it is plausible that the *YABBY14* gene regulates growth and development in transgenic *Arabidopsis* via ABA and GA signaling pathways. To summarize, the study strongly suggests that *PvYABBY14* functions as a positive regulator. These research findings offer a solid foundation for comprehending the biological processes influenced by *YABBY* genes in plant growth and stress responses. Importantly, they hold valuable reference for potential applications in future production. Investigating the expression patterns of *YABBY* genes in plant growth, development, and stress responses has the potential to enhance stress resistance in switchgrass, with the ultimate goal of increasing biomass yield.

## Conclusion

In this investigation, we systematically analysed and classified YABBY members from switchgrass and characterized their expression patterns under normal growth, stress, and hormone treatments. The functional study of the *PvYABBY14* gene in *Arabidopsis* further explains the potential roles of the PvYABBY family members. These findings contribute valuable insights into the functions and regulatory mechanisms of PvYABBY proteins, particularly in the context of switchgrass growth, development, and abiotic stress tolerance.

## Materials and methods

### Identification and phylogenetic analysis

To identify the *PvYABBY* gene family members from the switchgrass genome database, the Hidden Markov Model profile of the YABBY domain (PF04690) obtained from the Pfam database (http://pfam.xfam.org/) was applied as a query to search for YABBY members. Confirmation of conserved YABBY domains among the candidate members was achieved through NCBI CD search [53]. The theoretical isoelectric point (pI) and molecular weight (MW) of all the confirmed PvYABBY proteins were determined using the ExPASy ProtParam tool (http://expasy.org/). All the YABBY members from *Arabidopsis* [10], rice [11], maize [13], soybean [15], and sorghum [24] were obtained from previous studies. Multiple sequence alignments of protein sequences were performed using ClustalX 2.1 [54]. The neighbor-joining method from MEGA7 was used to construct the unrooted phylogenetic tree of all the aligned fully predicted protein sequences (1000 bootstrap replications, a Poisson model, and pairwise deletion) [55]. Finally, the phylogenetic tree was visualized using the EvolView online programmer [56].

### Conserved motif and gene structure analysis

The conserved motifs of PvYABBY proteins were identified using the MEME server (Multiple Em for Motif Elicitation; https://meme-suite.org/tools/meme) with the following requirements: motif width = 6 < *n* < 100, maximum number of motifs =10. The intron/exon structure information of PvYABBY proteins was sourced from the JGI database. The resulting integrated schematic, encompassing the phylogenetic tree, conserved domains, and gene structures, was visualized using the EvolView online platform.

### Synteny analysis

The positions of the *YABBY* genes were extracted from JGI database (https://phytozome-next.jgi.doe.gov/) [57]. MCscanX was employed to analyse the synteny links using the gene positions as anchors [58]. Then, the collinear gene pairs of the *PvYABBY* family were visualized by Circos software (v.0.69) [59]. Tandem duplication events were defined as two or more neighboring genes on the same chromosome. Segmental duplication events were defined as duplicate pairs located on different chromosomes [60].

### Expression pattern analysis of different tissues

The expression patterns of the *PvYABBY* genes in various tissues were downloaded from JGI database. The expression profile of *PvYABBY* genes was visualized using R software (V. 4.3.0).

### Promoter analysis

The 1500 bp upstream regions of the initiation codon (ATG) of *YABBY* genes were extracted from the *P. virgatum* genome. The PlantCare database (http://bioinformatics.psb.ugent.be/webtools/plantcare/html/) was employed to investigate the promoter region to predict putative cis-acting elements. The results were analysed and visualized using Microsoft Excel 2016.

### Plant materials and treatments

After seven days of germination on filter paper, plump switchgrass (cv. Alamo) seeds were carefully selected and transferred to a hydroponic nutrient solution for continued growth. The growth conditions were maintained at 28 °C with 14 h of light and 23 °C with 10 h of darkness. Stresses and hormones, including salt (NaCl, 200 mmol·L^−1^), drought (PEG, 20% w/v), ABA (50 μmol·L^−1^) and GA (100 μmol·L^−1^), were applied after 30 days of growth (5-leaf stage) and sampled at 0 h, 2 h, 6 h and 12 h. The inverted second leaves were sampled and quickly frozen in liquid nitrogen and stored in an − 80 ultralow temperature refrigerator.

### RNA extraction, cDNA synthesis and qRT–PCR analysis

According to the manufacturer’s instructions, RNAiso Plus (TaKaRa, Dalian, China) was used to extract total RNA, and cDNA synthesis was carried out with the PrimeScript™ RT Reagent Kit with gDNA Eraser (Perfect Real Time, TaKaRa, Dalian, China). qRT–PCR was carried out with the QuantStudio 5 Real-Time PCR system (Thermo Fisher, MA, USA) using TB Green® *Premix Ex Taq*™ (Tli RNaseH Plus, TaKaRa, Dalian, China). qRT-PCR program included initial denaturation at 95 °C for 10 minutes, followed by 40 cycles of denaturation at 95 °C for 5 seconds, and annealing/extension at 60 °C for 31 seconds. All PvYABBY primers were designed by Primer 6 software and blast in the JGI database to confirm the specificity. Elongation factor 1α (EF1α) was employed as the reference gene for the calculation of the expression level. Each biological procedure was repeated in triplicate to confirm the accuracy of the results. The expression of *PvYABBY* genes was calculated by the 2^–ΔΔCt^ method and graphically screened on the bar chart with Microsoft Excel 2016 [61].

### Subcellular localization of *PvYABBYs*

The full-length coding sequence of *PvYABBYs* without a terminator codon (TAG) was cloned into the GFP vector to generate a *PvYABBYs*-GFP fusion protein for investigating subcellular localization. To introduce these constructs into *Nicotiana tabacum* plants, both 35S::*PvYABBYs*-GFP and *Nuclear*-mCherry marker construction (Guo et al., 2015) were co-transformed using *Agrobacterium tumefaciens* strain GV3101.

### Acquisition and phenotypic observation of transgenic plants

*PvYABBY14* was amplified through PCR using the following primers: 5′- ACAGCCCAAGCTACGCGTCTCGAGGCAAGGTGATCGTGAGGAATGT − 3′ (*XhoI* site) and 5′- CTGGTGATTTCAGCGAATTATCTAGAGAGGTACTTATTCAGTGCTCGTGAT − 3′ (*XbaI* site). Subsequently, the plasmid was subcloned into the pGreenII-OE plant expression vector harboring the 35S promoter. The recombinant vector was transformed into wild-type *Arabidopsis* (Columbia-0) via *Agrobacterium tumefaciens* (GV3101-psoup) mediated by the floral dip method [62].

To characterize the stress tolerance and hormone sensitivity of *PvYABBY14* transgenic *Arabidopsis*, homozygous overexpression plants (T4) and Col-0 (CK) were used in this study. The seeds were germinated on 1/2-strength Murashige-Skoog (MS) medium containing 1% sucrose and 0.75% agar with a growth temperature of 23 °C/21 °C (day/night) and a photoperiod of 16 h/8 h (day/night) after 3 days of low temperature stratification. After 5 days, the seedlings were carefully transferred to different treatment media (mannitol, 100 mmol·L^−1^ and 200 mmol·L^−1^; ABA, 5 μmol·L^−1^ and 20 μmol·L^−1^; GA, 50 μmol·L^−1^ and 100 μmol·L^−1^). The root length of seedlings treated for 10 days was measured, and the data were imported into Microsoft Excel 2016 for processing.

### Statistical analysis

The statistical analyses were performed with SPSS 26.0 employing one-way ANOVA, Statistically significant differences between treatments were determined at *P* < 0.05 or *P* < 0.01.

### Supplementary Information

**Additional file 1: Supplementary Fig. 1.** A total of 10 sequence logos of motif1–10 found by Meme program. **Supplementary Fig. 2.** The transcript level of *PvYABBY14* displayed by the full-length gel using seq RT–PCR. **Supplementary Fig. 3.** Phenotypic comparison of wild-type (WT) and *PvYABBY14* transgenic *Arabidopsis* under ABA and PEG treatments. (a) Seedlings grown on 1/2 MS medium supplemented with 20 μmol·L^−1^ ABA. (b) Seedlings grown on 1/2 MS medium supplemented with 100 mmol·L^−1^ Mannitol. (c) Seedlings grown on 1/2 MS medium supplemented with 200 mmol·L^−1^ Mannitol. Scale bar = 1 cm.

**Additional file 2: Supplementary Table 1.** Summary of PvYABBY proteins from *Arabidopsis*, rice, maize, soybean, sorghum and switchgrass. **Supplementary Table 2.** Synteny information of Pvyabby genes in switchgrass. **Supplementary Table 3.** Synteny pairs of YABBY genes among *Panicum virgatum* with *Arabidopsis thaliana*, *Glycine max*, *Oryza sativa*, *Zea mays*, *Sorghum bicolor*. **Supplementary Table 4.** Predicted cis-elements associated with hormone and stress responses in Pvyabby gene promoters. **Supplementary Table 5.** Transcript levels of *PvYABBY* genes in switchgrass. **Supplementary Table 6.** Primers used in qRT-PCR.

## Data Availability

The datasets generated and/or analyzed during this study are available in Joint Genomics Institute database (https://phytozome-next.jgi.doe.gov/) and the National Centre for Biotechnology Information database(http://www.ncbi.nlm.nih.gov/)with accession no. PRJNA40657 and PRJNA704030. Other data are available in the Supplementary files. Genome files of *Panicum virgatum*, *Arabidopsis thaliana*, *Oryza sativa*, *Glycine max*, *Zea mays*, *Sorghum bicolor* species were obtained from Phytozome (https://phytozome-next.jgi.doe.gov/).
