# Supplementary material for: Genome-wide analysis of the switchgrass YABBY family and functional characterization of PvYABBY14 in response to ABA and GA stress in Arabidopsis
Source: BMC Plant Biol. 2024 Feb 16;24:114. doi: 10.1186/s12870-024-04781-7 (PMC10870668; doi:10.1186/s12870-024-04781-7)
Supplement: Supplementary file 1 — Additional file 1: Supplementary Fig. 1. A total of 10 sequence logos of motif1–10 found by Meme program. Supplementary Fig. 2. The transcript level of PvYABBY14 displayed by the full-length gel using seq RT–PCR. Supplementary Fig. 3. Phenotypic comparison of wild-type (WT) and PvYABBY14 transgenic Arabidopsis under ABA and PEG treatments. (a) Seedlings grown on 1/2 MS medium supplemented with 20 μmol·L−1 ABA. (b) Seedlings grown on 1/2 MS medium supplemented with 100 mmol·L−1 Mannitol. (c) Seedlings grown on 1/2 MS medium supplemented with 200 mmol·L−1 Mannitol. Scale bar = 1 cm. [file 12870_2024_4781_MOESM1_ESM.pdf]

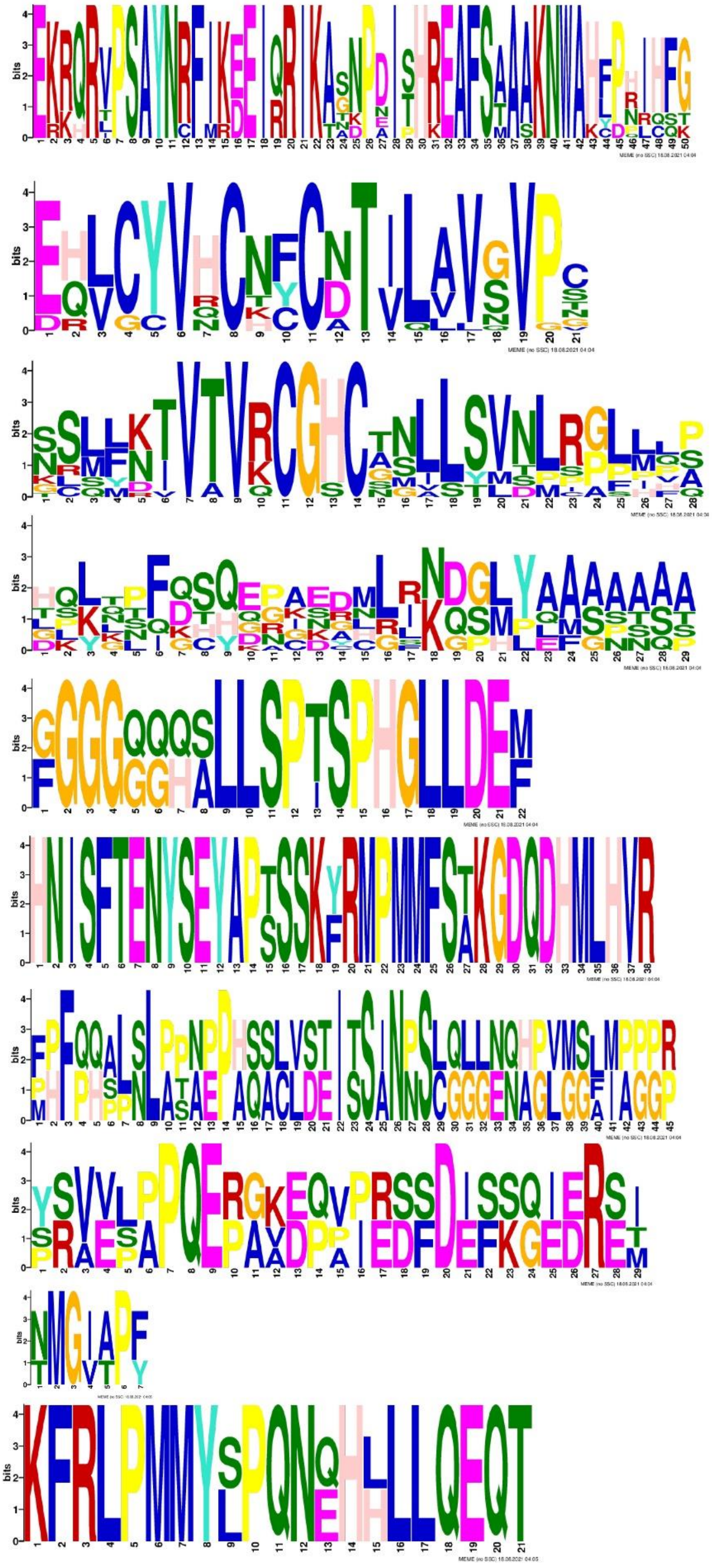

Supplementary figure 1 A total of 10 sequence logos of motif1-10 found by Meme program

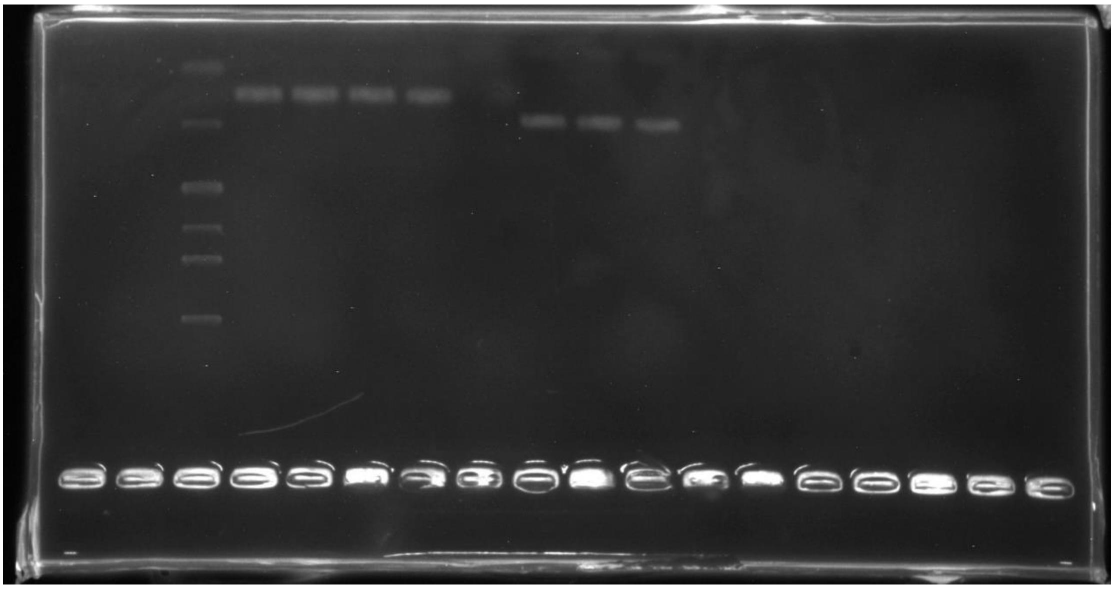

Supplementary figure 2 The transcript level of *PvYABBY14* displayed by the full-length gel using seq RT–PCR

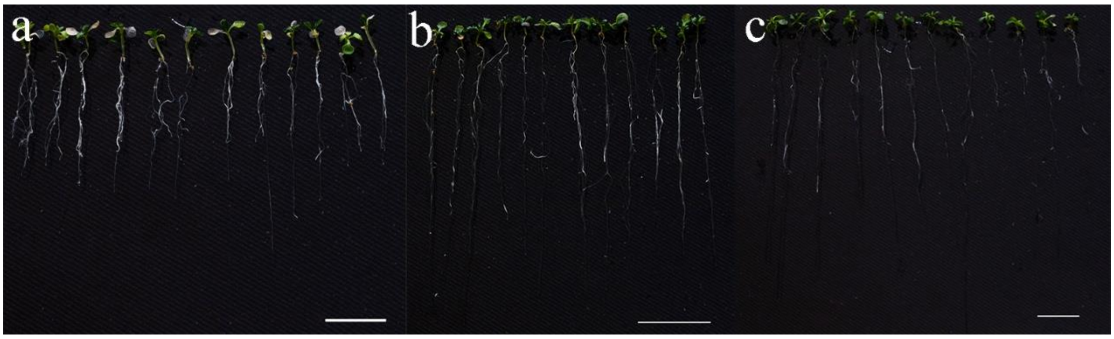

Supplementary figure 3 Phenotypic comparison of wild-type (WT) and *PvYABBY14* transgenic Arabidopsis under ABA and PEG treatments. (a) Seedlings grown on 1/2MS medium supplemented with 20 μmol·L^{-1} ABA. (b) Seedlings grown on 1/2 MS medium supplemented with 100 mmol·L^{-1} Mannitol. (c) Seedlings grown on 1/2MS medium supplemented with 200 mmol·L^{-1} Mannitol. Scale bar=1cm
